# Supplementary material for: Kinesin-1 regulates antigen cross-presentation through the scission of tubulations from early endosomes in dendritic cells
Source: Nat Commun. 2020 Apr 14;11:1817. doi: 10.1038/s41467-020-15692-0 (PMC7156633; doi:10.1038/s41467-020-15692-0)
Supplement: Supplementary file 1 — Supplementary Information [file 41467_2020_15692_MOESM1_ESM.pdf]

Supplementary Information

Kinesin-1 regulates antigen cross-presentation through the scission of tubulations from early endosomes in dendritic cells

Belabed et al.

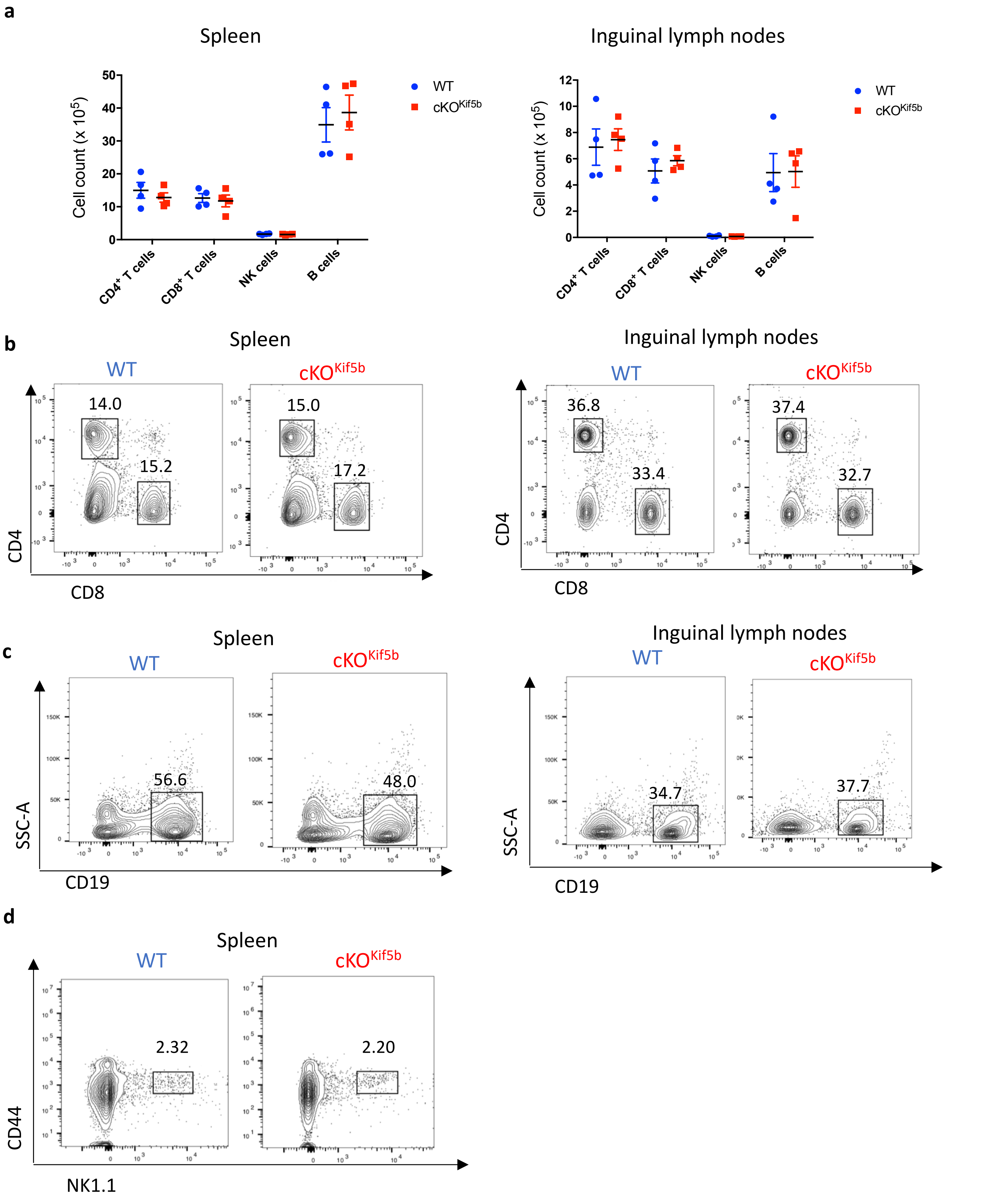

Supplementary Figure 1: cKO^{Kif5b} display normal numbers of the lymphoid lineage. **a** Absolute numbers of CD4^{+} T cells, CD8^{+} T cells, NK cells and B cells in the spleen and the inguinal lymph nodes of WT (blue circle) and cKO^{Kif5b} (red square) mice at the steady state. The data are representative of four independent experiments. Graphs show mean ± S.E.M.. **b** Contour plots of T cells (CD4^{+} and CD8^{+}) from the spleen and the inguinal lymph nodes of either WT or cKO^{Kif5b} mice at the steady state. **c** Contour plots of B cells (CD19^{+}) from the spleen and the inguinal lymph nodes of either WT or cKO^{Kif5b} mice at the steady state. **d** Contour plots of NK cells (NK1.1^{+}, CD44^{+}) from the spleen of either WT or cKO^{Kif5b} mice at the steady state.

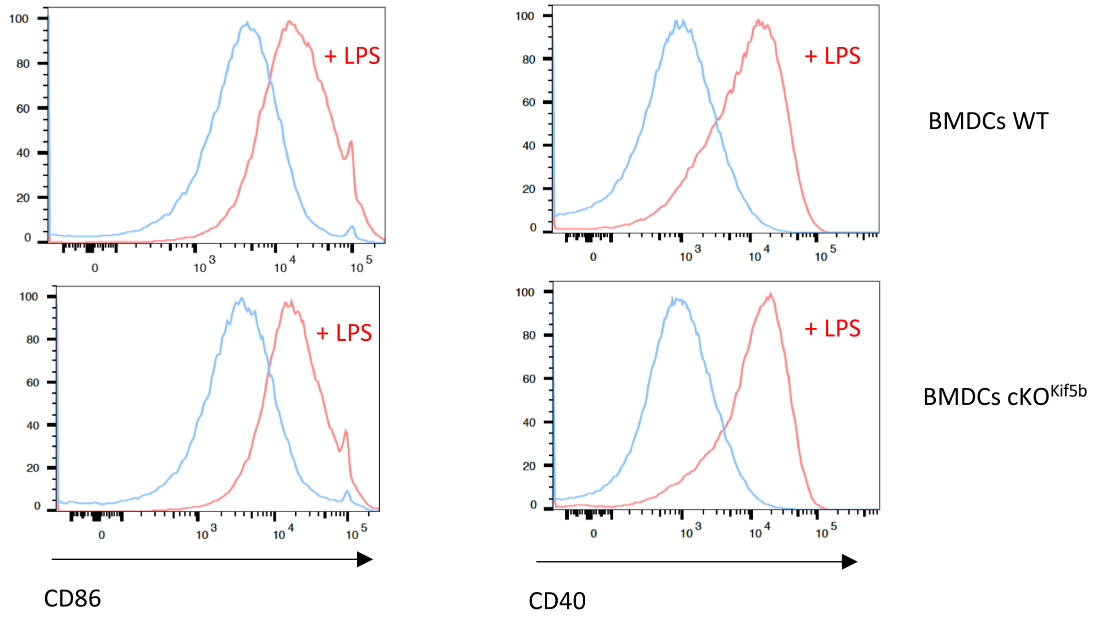

Supplementary Figure 2: cKO^{Kif5b} BMDCs are normally activated by LPS. Representative flow cytometry of CD86 and CD40 expression by BMMCs from WT or cKO^{Kif5b} mice. Cells were activated for four hours with 1 μg/mL lipopolysaccharide (LPS)(red line).

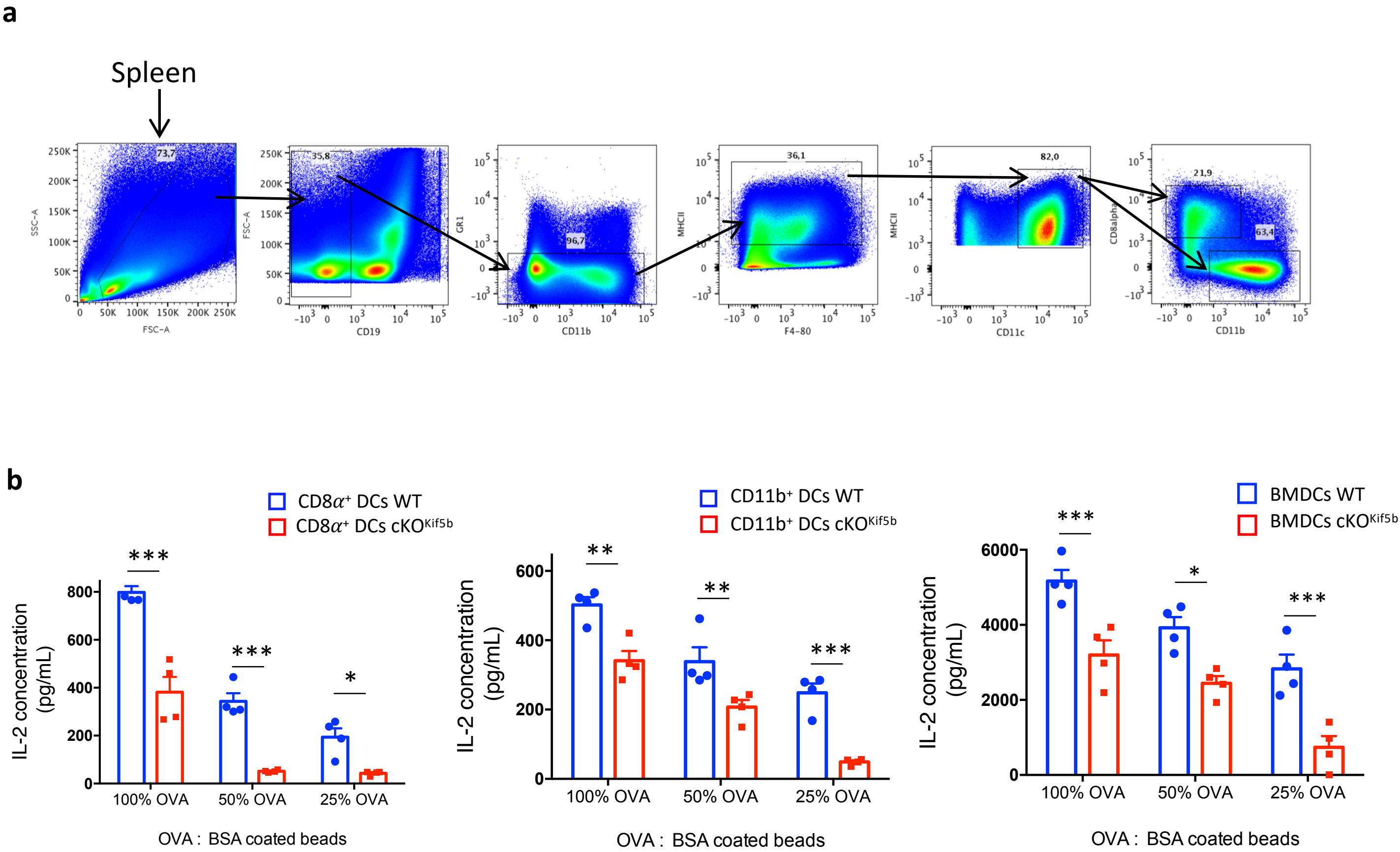

Supplementary Figure 3: Kinesin-1 deficiency impairs cross-presentation of particulate Ag. **a** Gating strategy for purification of CD8α^{+} and CD11b^{+} DCs from the spleen. **b** Cross-presentation efficiency of CD8α^{+} DCs, CD11b^{+} DCs and BMDCs from WT (blue histogram) or cKO^{Kif5b} (red histogram) mice in the presence of different concentration of bead-bound OVA, measured as IL-2 secretion by OT-I T cells after 16 hours of co-culture. The data are representative of four independent experiments. Graphs show mean ± S.E.M.. Statistical analysis: *, P<0.05; **, P<0.005; ***, P<0.0001 in a two-way ANOVA and Sidak test's correction for multiple comparison.

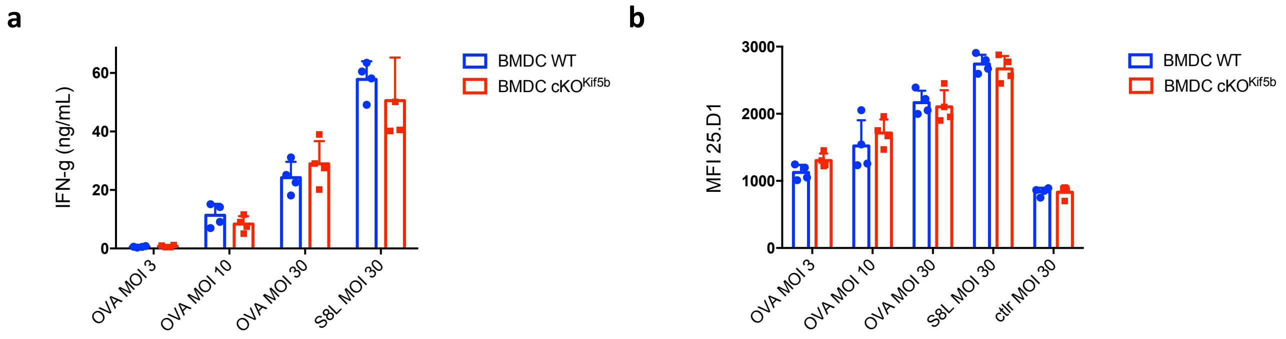

Supplementary Figure 4: cKO^{Kif5b} BMDCs did not affect direct presentation of intracellular OVA. a BMDCS from WT (blue histogram) or cKO^{Kif5b} (red histogram) mice were infected by vaccinia virus expressing OVA or the H-2K^{b} restricted epitope SIINFEKL (S8L) or the control strain (CTRL), and mixed with OT-I T cells (ratio: 2:1) to assess direct Ag presentation. Secretion of IFN-γ by OT-I cells was measured in an ELISA. The data are representative of four independent experiments. **b** Formation of S8L/H2-Kb complexes at the cell surface of BMDC WT (blue histogram) and cKO^{Kif5b} (red histogram) evaluated 6 h after infection with vaccinia viruses expressing OVA or peptide SIINFEKL (S8L) or with the control strain (CTRL), as assessed by staining cells with the 25-D1.16 monoclonal antibody; the values correspond to the MFI for 25-D1.16. The data are representative of four independent experiments. Graphs show mean ± S.E.M.. No statistical difference was found in a two-way ANOVA and Sidak test's correction for multiple comparison.

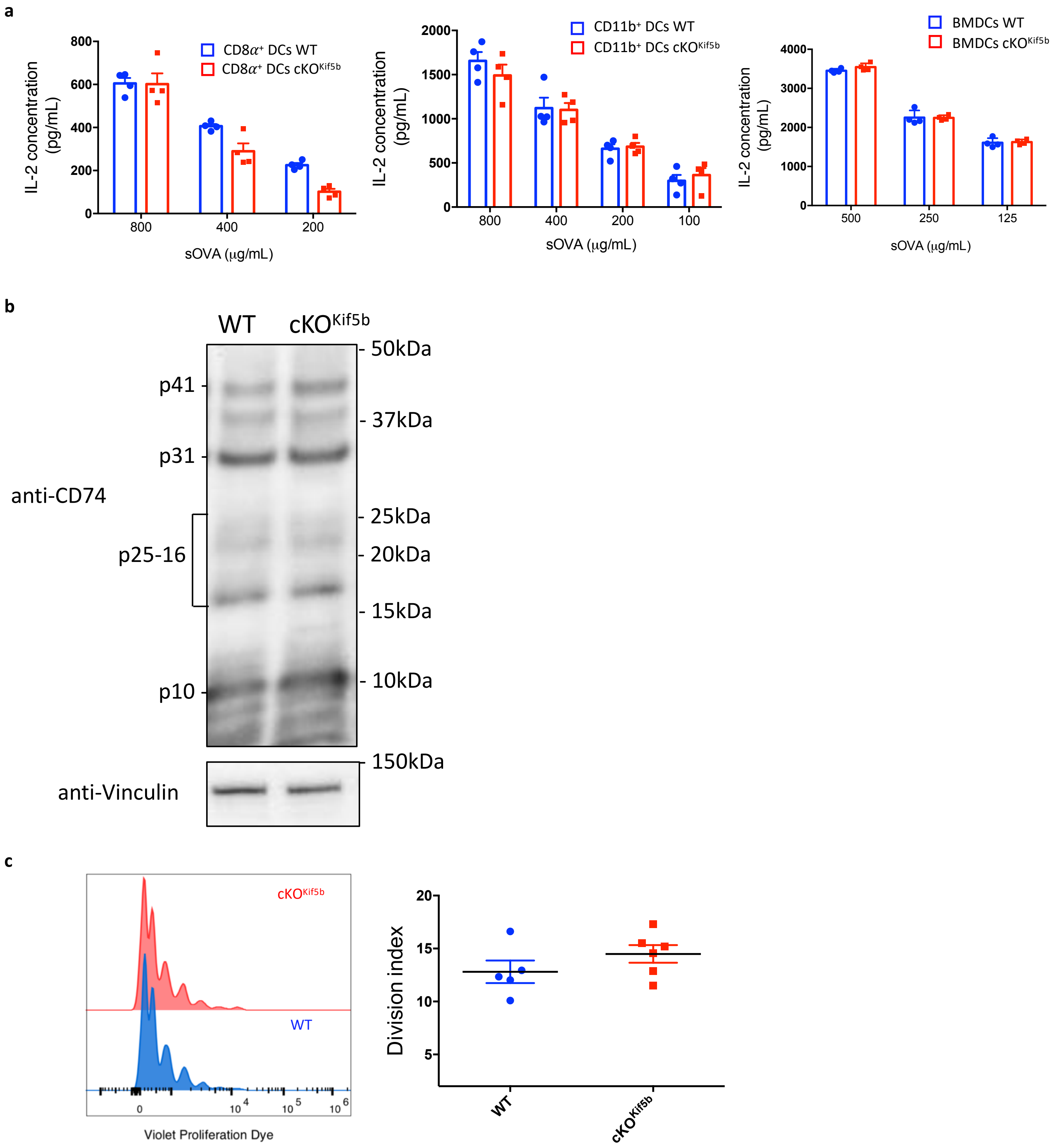

Supplementary Figure 5: Kinesin-1 deficiency does not impair MHC-II presentation. a The MHC-II presentation efficiency of CD8α$^+$ DCs, CD11b$^+$ DCs and BMDCs from WT (blue histogram) or cKO^{Kif5b} (red histogram) mice in the presence of different concentrations of sOVA, measured as IL-2 secretion by OT-II T cells after 16 hours of co-culture. The data are representative of at least three independent experiments. No statistical difference was found in a two-way ANOVA and Sidak test's correction for multiple comparison. **b** WT and cKO^{Kif5b} BMDC lysates were separated by SDS-PAGE and immunoblotted with anti-CD74 (BD biosciences) and anti-Vinculin (ThermoFisher) antibodies. One experiment representative out of two is shown. **c** Mice (WT blue line or circles, cKO^{Kif5b} red line or circles)(WT mice n=5, cKO^{Kif5b} mice n=6) were injected with Violet-labelled transgenic OT-II T cells and primed 1 day later with CD11c/P3UOVA. The left panel shows representative profiles gated on CD4$^+$, TCR Vβ5.1/5.2$^+$ cells. The division index is shown in the right panel. No statistical difference was found in a two-tailed unpaired student's *t* test. **a**-**c** Graphs show mean ± S.E.M..

Supplemental Figure 5b

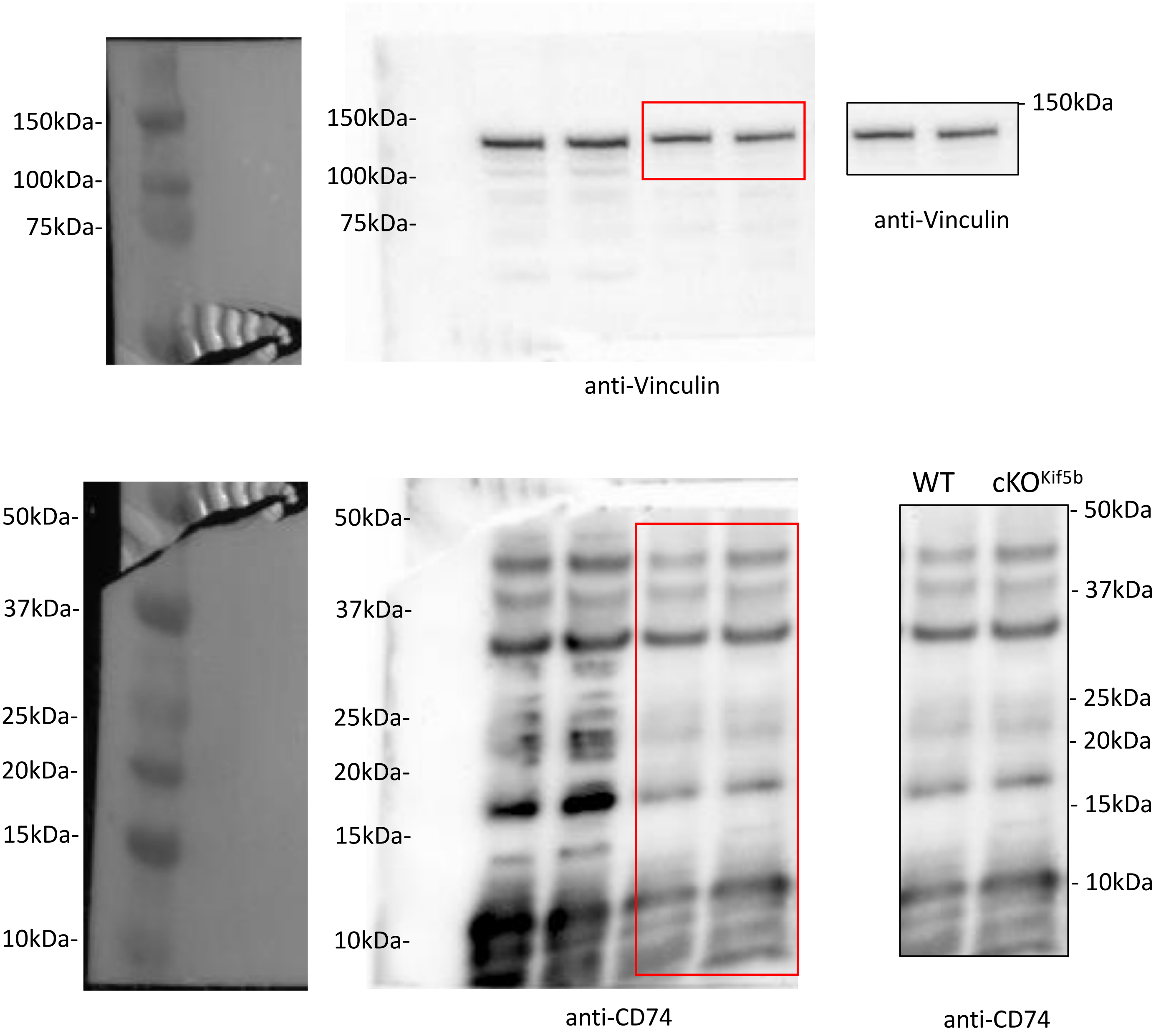

Uncropped immunoblots from supplementary figure 5b

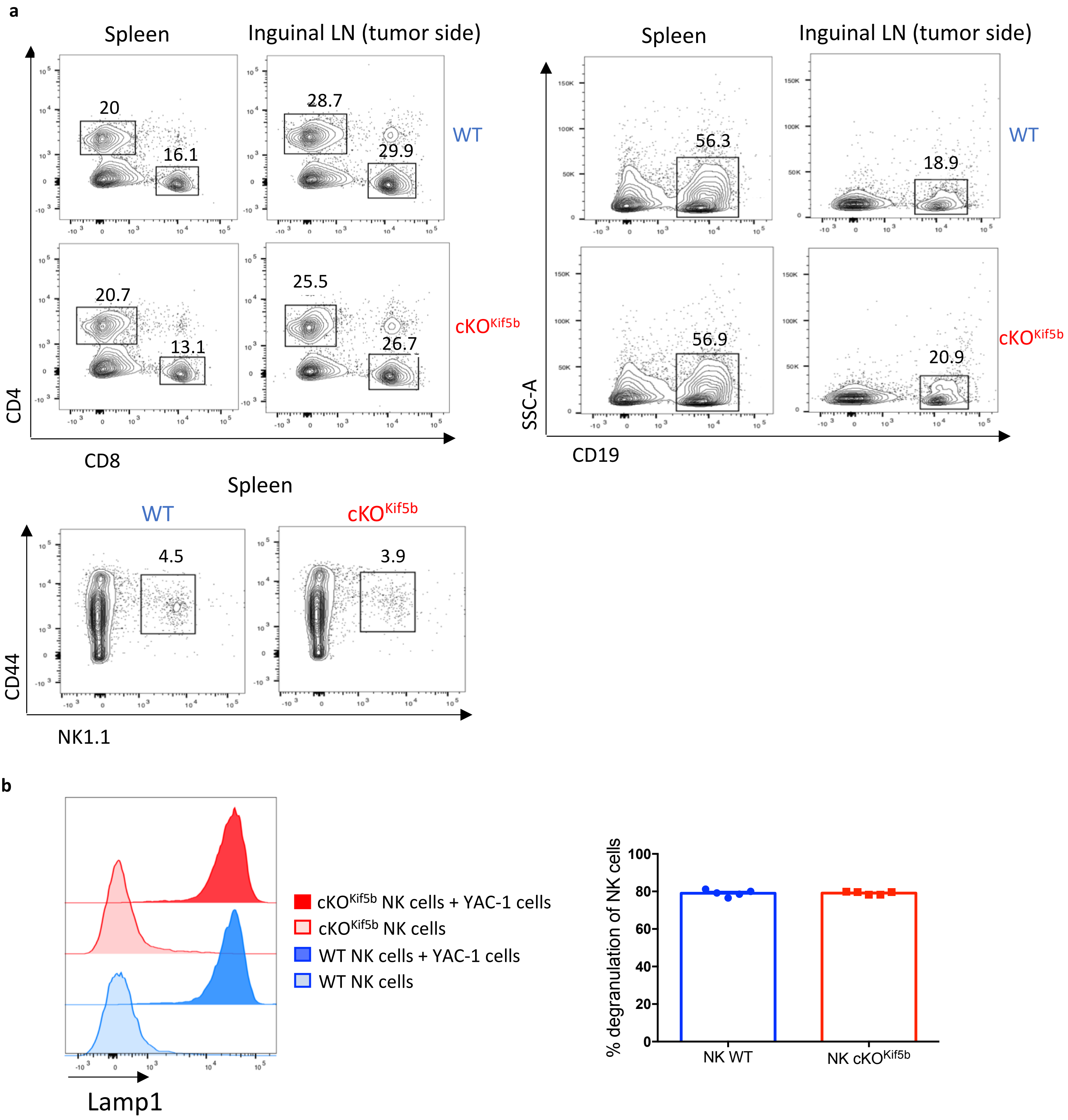

Supplementary Figure 6: Ten days after tumour injection, immune populations are present in normal numbers and NK cells are functional in cKO^{Kif5b}. a Contour plots of T cells (CD4$^+$ and CD8$^+$), B cells (CD19$^+$) and NK cells (NK1.1$^+$, CD44$^+$) from the spleen and the inguinal lymph nodes of WT or cKO^{Kif5b} mice ten days after the injection of B16-OVA are shown. **b** NK cells from WT (Blue line or histogram) and cKO^{Kif5b} (red line or histogram) mice were co-cultured or not with YAC-1 cells at an E:T ratio of 1:3. Representative flow cytometry profiles of NK cell (gated on NK1.1$^+$) CD107a (Lamp1) expression are shown (Left panel). The data are representative of 3 independent experiments. The percentage of degranulation (Lamp1$^+$ NK cells) of NK cells from WT and cKO^{Kif5b} mice co-cultured with YAC-1 cells is shown in the right panel. Graph shows mean ± S.E.M..

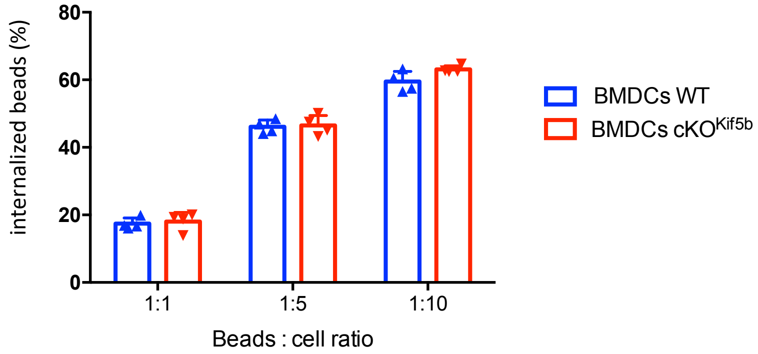

Supplementary Figure 7: Kinesin-1 does not regulate phagocytotic efficiency. The phagocytotic efficiency of BMDCs from WT (blue histogram) or cKO^{Kif5b} (red histogram) mice is measured using FACS. The internalization of 3 µm latex beads was measured at pH 4 with trypan blue (TB). The data are representative of four independent experiments. Graph shows mean ± S.E.M..

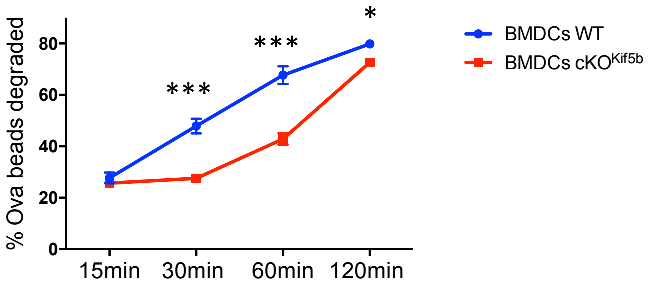

Supplementary Figure 8: Kinesin-1 regulates Ag degradation of particulate Ag. Ag bead degradation by BMDCs from WT (blue line) or cKO^{Kif5b} (red line) mice, measured using FACS. BMDCs were pulse-chased with coupled latex beads at the indicated time points. The data are representative of eight independent experiments. Statistical analysis: *, $P<0.05$; ***, $P<0.0001$ in a two-way ANOVA and Sidak test's correction for multiple comparison. Graph shows mean ± S.E.M..

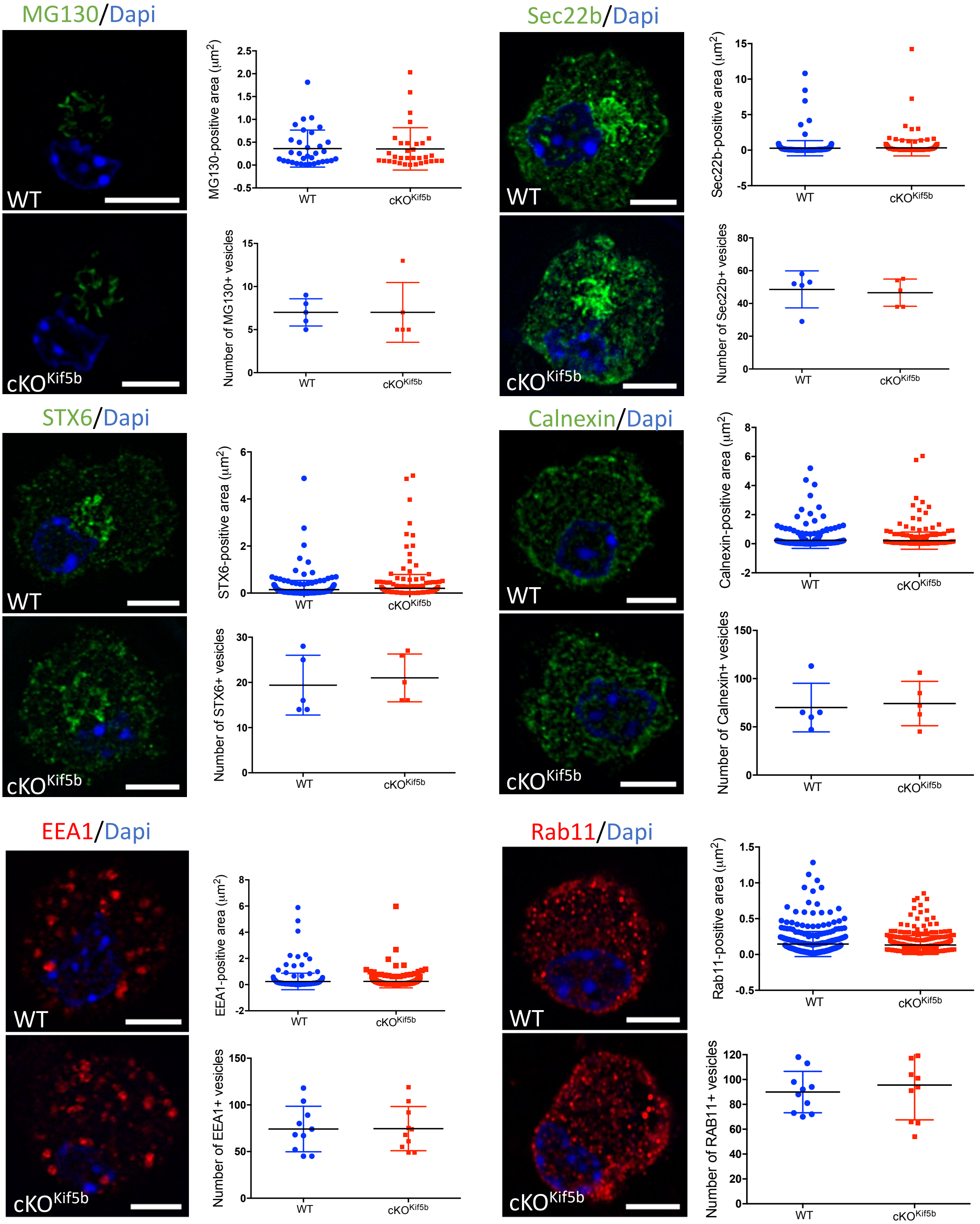

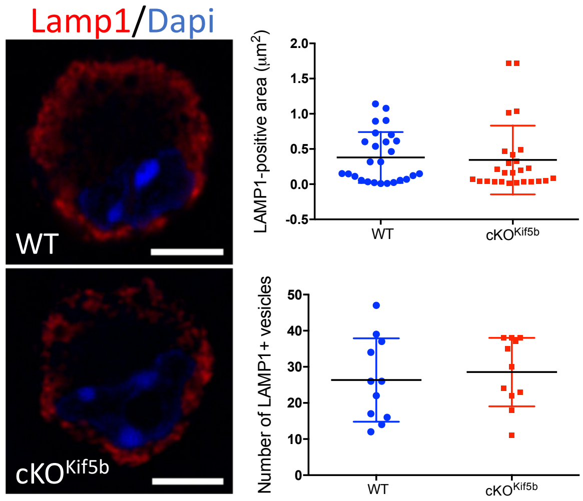

Supplementary Figure 9: The number of vesicles and morphology of the different endosomal compartments, Golgi apparatus and ER have remained unaltered in cKO^{Kif5b} BMDCs. Confocal microscopy of BMDCs from WT or cKO^{Kif5b} mice. Cells were plated on glass coverslips, fixed, permeabilized, and stained with anti-MG130, anti-Sec22, anti-STX6, anti-calnexin, anti-EEA1, anti-Rab11 or anti-Lamp1 antibodies. Bars: 5 µm. All images of single cells are representative of >100 cells observed in at least three independent experiments. Quantification of the number of marker-positive vesicles or the marker-positive area is shown on the right of each image (WT blue histogram, cKO^{Kif5b} red histogram). Graphs show mean ± S.E.M..

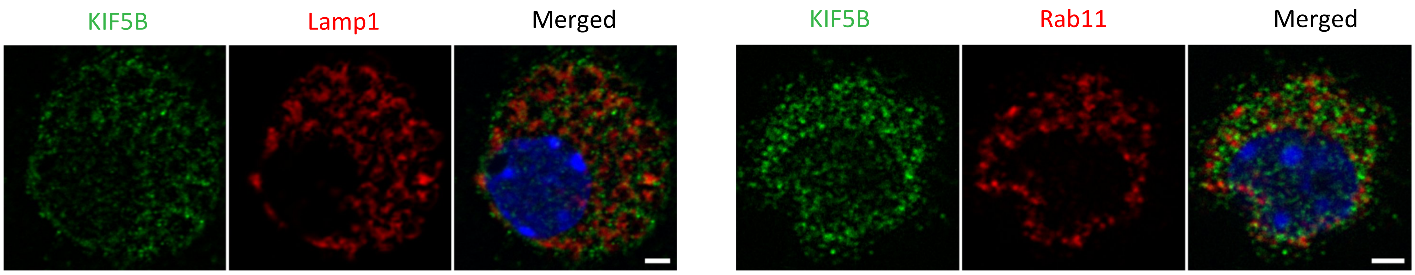

Supplementary Figure 10: Kif5b colocalization with Lamp1 or Rab11. Cells were plated on glass coverslips, fixed, permeabilized, and stained with anti-Lamp1, anti-Rab11 or anti-Kif5b antibodies. Bars: 2 μm. All images of single cells are representative of >100 cells observed in at least three independent experiments.

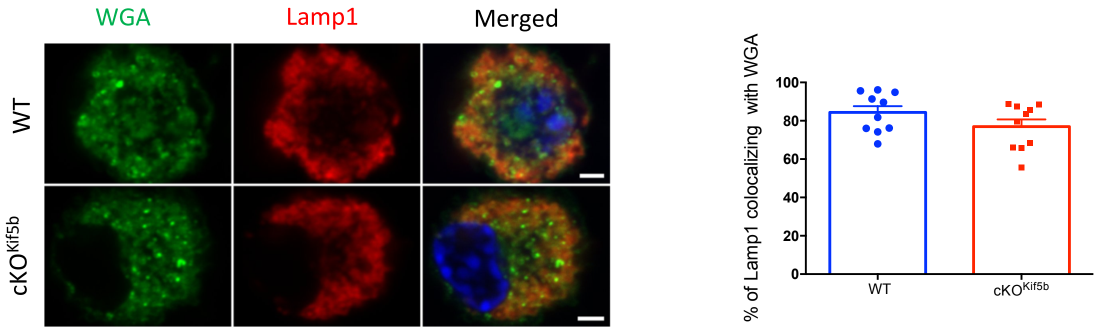

Supplementary Figure 11: Confocal microscopy of WT and cKO^{kif5b} BMDCs pulsed for 5 min with WGA-488 and chased for 2h. Cells were then fixed, permeabilized, and stained with an anti-Lamp1 antibody. Bars: 2 μm. All images of single cells are representative of >100 cells observed over at least three independent experiments (left panel). Quantification of WGA-488/Lamp1-555 colocalization (right panel). Graph shows mean ± S.E.M..

a

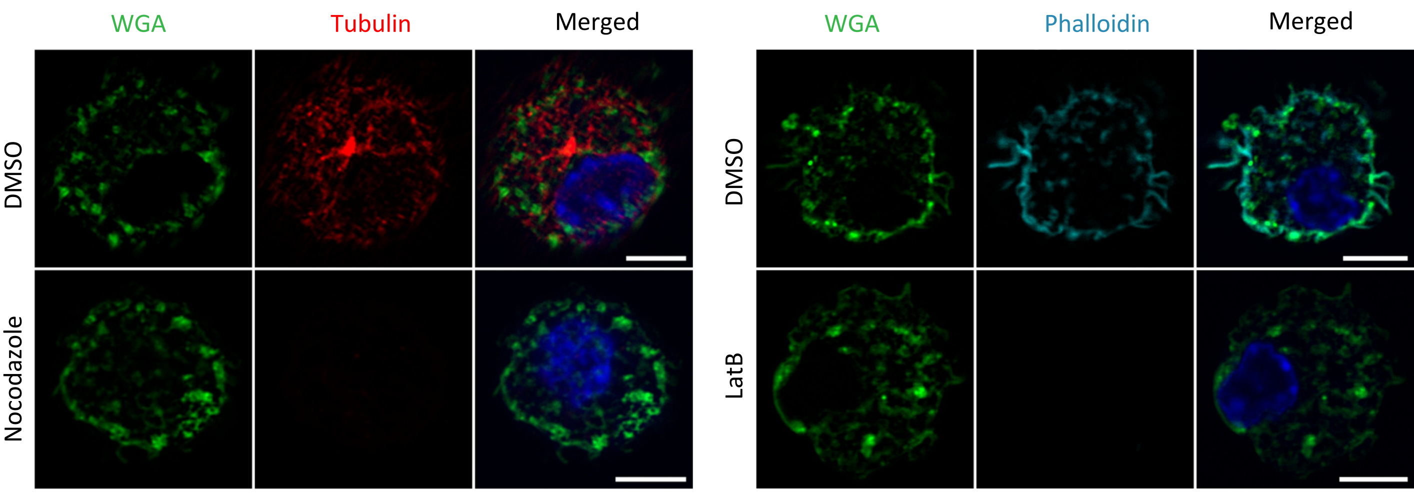

b

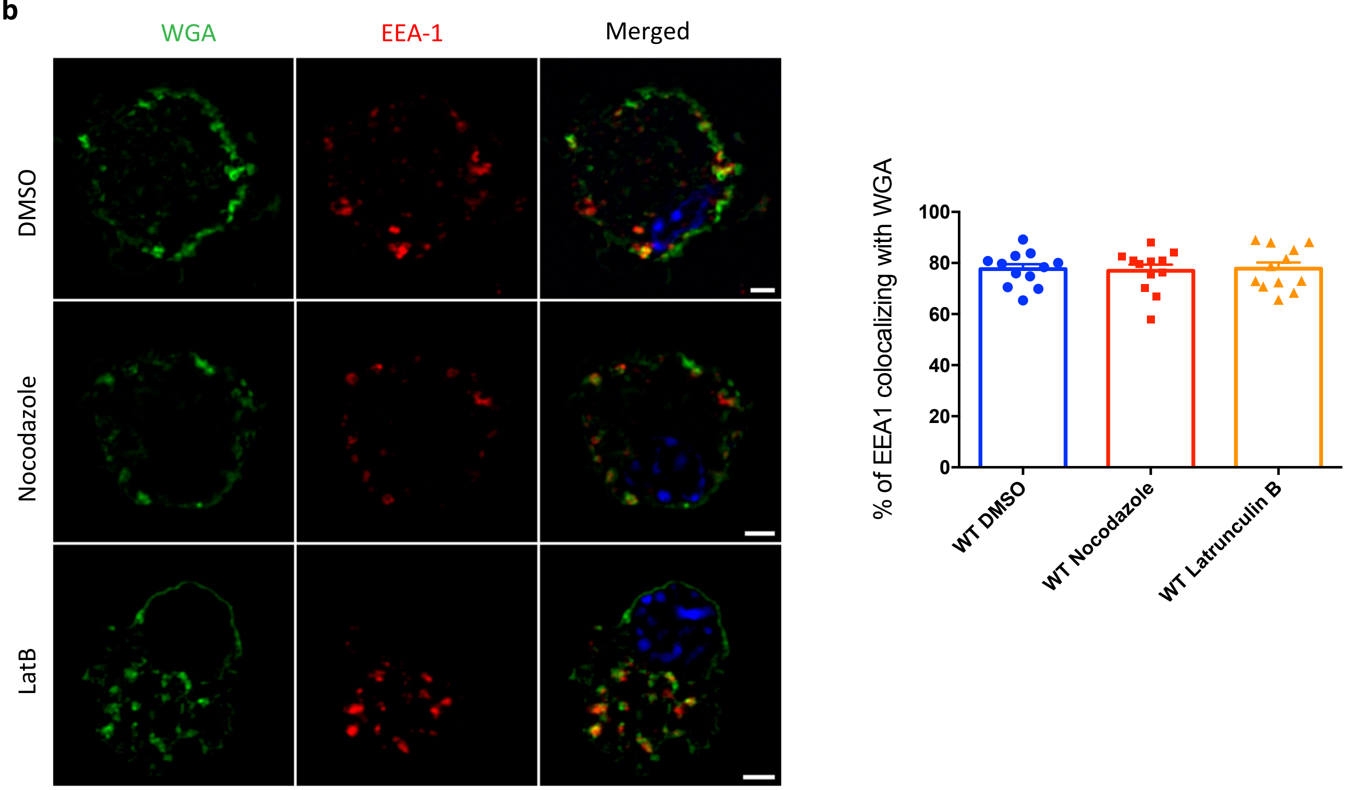

c

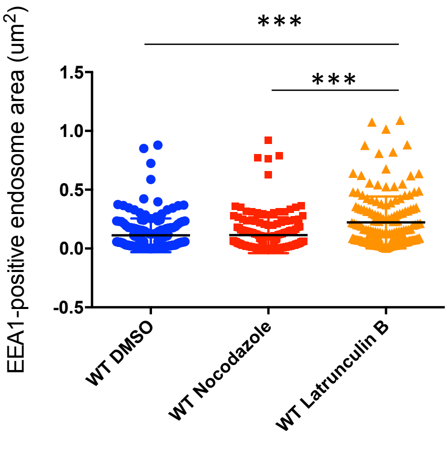

Supplementary Figure 12: Effect of nocodazole or LatB on cytoskeleton and WGA internalization. a Confocal microscopy of WT BMDCs pre-treated with DMSO, nocodazole or LatB, pulsed for 5 min with WGA-488, and chased for 5 min. Cells were plated on glass coverslips, fixed, permeabilized, and stained with anti-tubulin antibody or phalloidin. Bars: 5 μm. **b** WT BMDCs pre-treated with DMSO (blue histogram), nocodazole (red histogram) or LatB (yellow histogram) were labelled with WGA-488 and plated on glass coverslips. Cells were then fixed, permeabilized, and stained with anti-EEA1 antibody. Bars: 2 μm (left panel). Quantification of WGA-488/EEA1-555 colocalization (right panel). All images of single cells are representative of >100 cells observed over at least three independent experiments (**a**, **b**). **c** The size of the EEA1-positive vesicular structures in WT BMDCs pre-treated with DMSO (blue circle), nocodazole (red square) or LatB (yellow triangle) was least observed in three independent experiments. Statistical analysis: ***, P<0.0001 in a one-way ANOVA and Dunn's test's correction for multiple comparison. **b-c** Graphs show mean ± S.E.M..

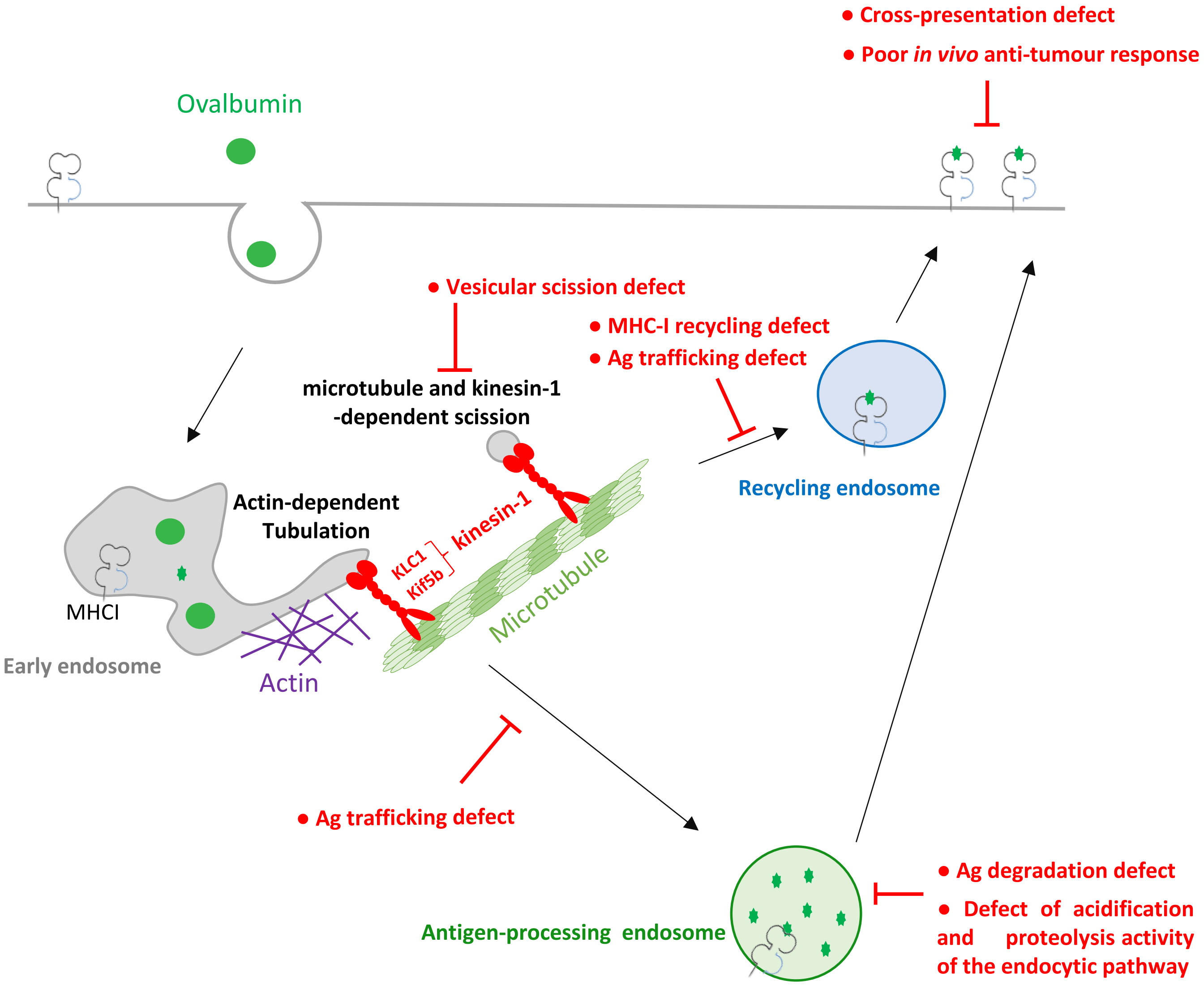

Supplementary Figure 13: Role of kinesin-1 in early endosome dynamics and antigen cross-presentation. The characterization of DCs or BMDCs from cKO^{Kif5b} mice has highlighted a kinesin-1 function in Ag degradation, acidification and proteolysis activity in the endocytic pathway, and MHC-I recycling by regulating the early endosome dynamics. Kinesin-1 appears to regulate early endosome maturation by allowing the scission of endosomal tubulations. All cellular defects caused by the absence of Kif5b are listed in red on the scheme.
